# The Alterations in the Brain Corresponding to Low Back Pain: Recent Insights and Advances

**DOI:** 10.1155/2024/5599046

**Published:** 2024-03-18

**Authors:** Xuyang Li, Fancheng Meng, Wenye Huang, Yue Cui, Fanbo Meng, Shengxi Wu, Hui Xu

**Affiliations:** ^1^Department of Neurobiology and Collaborative Innovation Center for Brain Science, School of Basic Medicine, The Fourth Military Medical University, Xi'an, China; ^2^College of Life Sciences, Northwest University, Xi'an, China

## Abstract

Low back pain (LBP) is a leading cause of global disabilities. Numerous molecular, cellular, and anatomical factors are implicated in LBP. Current issues regarding neurologic alterations in LBP have focused on the reorganization of peripheral nerve and spinal cord, but neural mechanisms of exactly what LBP impacts on the brain required further researches. Based on existing clinical studies that chronic pain problems were accompanying alterations in brain structures and functions, researchers proposed logical conjectures that similar alterations occur in LBP patients as well. With recent extensive studies carried out using noninvasive neuroimaging technique, increasing number of abnormalities and alterations has been identified. Here, we reviewed brain alterations including white matters, grey matters, and neural circuits between brain areas, which are involved in chronic LBP. Moreover, brain structural and functional connectivity abnormalities are correlated to the happening and transition of LBP. The negative emotions related to back pain indicate possible alterations in emotional brain regions. Thus, the aim of this review is to summarize current findings on the alterations corresponding to LBP in the brain. It will not only further our understanding of etiology of LBP and understanding of negative emotions accompanying with back pain but also provide ideas and basis for new accesses to the diagnosis, treatment, and rehabilitation afterward based on integral medicine.

## 1. Introduction

Chronic back pain (CBP) is one of the major health issues worldwide. Recent studies estimated the direct and indirect costs of back pain up to $624.8 billion [1]. And, unlike other chronic painful disorders, neither the nature nor the impact of brain changes in response to back pain is well understood. A variety of treatments have not been tested and proved effective at reducing pain and disability due to some of these changes. Therefore, it is urgent and pressing for CBP to dig a deep understanding of its causes and outcomes in clinic. As more and more researchers concentrated on it, several novel findings have been obtained through the development of noninvasive neuroimaging techniques in this field.

Lumbar disc degeneration is considered playing a primary role in back pain, particularly in CBP. It has been fully proven that disc degeneration resulting in neurotrophins and inflammatory mediators [2, 3], including interleukin-1, brain-derived neurotrophic factor, and nerve growth factors, contributed to the pathogenesis of back pain [4–7]. So the “end organs disability,” of which muscle and skeleton abnormalities consist, were focused in clinical practice. Nevertheless, nearly 90% of patients with apparent symptoms can not obtain accurate diagnosis, and most patients were nonspecific, making it even harder to be diagnosed and treated, and thus, it led to the delay in treatment and aggravation of the condition [8].

It would be significant to evaluate abnormalities in the brain for a better understanding of causes and effects of the LBP [9, 10]. Especially given the fact that CBP patients were more likely to suffer from negative emotion (depression/anxiety/fear), according to a study mainly consisted of a questionnaire with 228 variables which was administered to 109 randomly selected patients. The result indicated that 63% exhibited clinically important levels of depression and 54% had clinically important levels of anxiety [11]. With the development of noninvasive neuroimaging techniques, extensive human and animal evidence indicated that LBP was associated with peripheral and central nervous system reorganization with a large list of neuronal and glial alterations. Currently, functional magnetic resonance imaging (fMRI) and electroencephalogram (EEG) stand were commonly used to investigate brain alterations in the field of chronic pain. It is noteworthy to point out these two ways share a set of common characteristics. Continuous EEG is able to record and characterize cortical electrophysiological responses during the generation and persistence of chronic pain. Previous EEG studies showed that theta, beta, and gamma frequency bands were associated with pain perception, in terms of the best rhythmic band correlating with different levels of pain. There was no clear consensus whether gamma waves were generated in deep brain areas, and therefore, it was difficult to measure with scalp EEGs. However, beta EEG activity associated with LBP was scarcely studied. Alpha band oscillations (8–13 Hz) were the most commonly explored and remained stable over time. Some studies showed that alpha frequency band was negatively linear with subjective perceived pain [12–17]. With EEG/MEG techniques, cortical changes could be measured with a high degree of temporal resolution and provided a deep insight into the dynamic process of pain information processing. Furthermore, fMRI techniques could provide massive spatial information related to cortical regions/networks involved in pain processing [18, 19]. fMRI has distinct advantages in terms of establishing the precise diagnoses of regions altered and follows up plan development. Thus, the alterations related to back pain, including the white matter, the grey matter, and a transition from subacute back pain (SBP; at least 1 month of persistent pain) to CBP (at least 1 year of persistent pain), and both were discussed from fMRI studies in the review. Potential future research directions and ideas about brain alterations of back pain were discussed as well.

## 2. Methods

This systematic review was reported following the PRISMA (Preferred Reporting Items for Systematic reviews and Meta-Analyses) guidelines.

## 3. Eligibility Criteria

The PICO approach was applied to formulate the key question: “What is the alterations in brain (O: outcome) in patients with CBP (P: populations) compared with healthy controls (C: comparison)?” This review was limited to studies that used fMRI (I: intervention) to examine the question. All types of interventions or exposures, tasks, or experimental paradigms performed to evoke certain fMRI responses were considered eligible.

## 4. Information Sources and Search Strategy

Studies that had been published in English in indexed and peer-reviewed journals between 1983 and May 2022 were included. About the criteria for considering studies for this review, we developed a search strategy to retrieve all relevant literature regarding this topic and published studies conducted on brain alterations corresponded to back pain in PubMed and Web of Science which were eligible for inclusion. A set of search terms was predefined based on the PICO question. P, I, and O were combined using the Boolean operator “OR.” P and I and P and O were combined with each other using the Boolean operator “AND.” The electronic databases of PubMed and Web of Science were uniformly searched between March 2021 and July 2022 with the following query: (“LBP” OR “Lumbar back pain” OR “lower back pain” OR “low back ache” OR “low backache” OR “lumbar pain” OR “lumbar spine pain”) AND (fMRI) NOT(EEG) OR ((“brain changes” OR “brain adaptations” OR “brain alterations” OR “brain function”) OR (“event related potential” OR “evoked potential”) AND (brain OR cortical)). It should be noted that the review studies found during the electronic search were not eligible to be included in the systematic review; however, their reference lists were also screened to identify potentially relevant studies.

## 5. Study Selection

Predefined in- and exclusion criteria regarding design, population, and topic of the studies were used to assess the eligibility of the search results (Table 1), during which titles and abstracts of the retrieved studies were screened to examine whether the studies met the inclusion criteria. If any of the inclusion criteria were not met, the study was excluded.

## 6. Qualification of Searchers/Raters

The review was searched, screened, and assessed for methodological quality by the first and second authors, and this was proceeded independently from each other. A comparison of the results from the search, screening on inclusion and exclusion criteria, and risk evaluation was conducted by these authors. Whenever there was a disagreement, the difference was discussed so that consensus could be reached. But when consensus could not be reached, a third opinion would be provided by the last author (H.X).

## 7. Grey Matter Alterations

**Figure 1:**
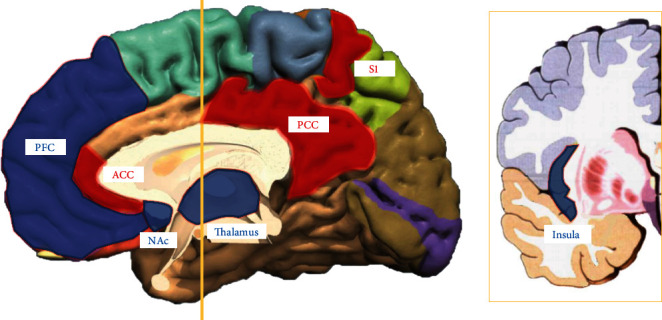
Structural alterations of grey matters in back pain patients. Increased changes of grey matters in red color, decreased changes of grey matters in blue color. ACC, anterior cingulate cortex; NAc, nucleus accumbens; PCC, posterior cingulate cortex; PFC, prefrontal cortex; S1, primary somatosensory cortex.

Some informative, noninvasive means of scanning and evaluating have been used in the studies, demonstrating brain abnormalities in patients with LBP, including structural abnormalities and regional activation alterations and functional connectivity alterations. We provided a review of recent studies that investigated brain alterations in LBP patients exhaustively in the following sections (Figure 1, Tables 2 and 3).

### 7.1. Structural and Regional Activation Alterations

A wealth of studies has demonstrated that many patients suffer from LBP showed cortical thickness and area changes in related brain regions [10]. By using structural MRI, resting-state fMRI, and task fMRI, and extracting morphological features from MRI via some advanced analysis techniques (e.g. voxel-based morphometry, VBM) [36], a number of anatomical differences in CBP patients have come into light over the observations. For example, the nucleus accumbens, the anterior cingulate cortex (ACC) [32, 33, 37] and the secondary somatosensory cortex (S2) were activated in the happening or transforming of back pain [38].

There have been many studies indicating that an increase in cortical thickness was common in patients with CBP [39]. In a study with 17 nonspecific low back pain (NSLBP) patients assessed, brain imaging analyses showed increased cortical thickness of the ventrolateral prefrontal cortical regions in patients with NSLBP compared to controls [20]. In another study including a total of 124 chronic low back pain (CLBP) patients, abnormalities in eight cortical regions including the left inferior parietal cortex, the left precentral gyrus, the left posterior cingulate cortex (PCC), the left middle temporal gyrus, the right rostral middle frontal gyrus, the right secondary sensorimotor cortex, and the right primary motor cortex. Among them, robust cortical thickness abnormalities were found in the left PCC and the right rostral middle frontal gyrus [21]. Nevertheless, some studies showed the opposite results. For example, some pain-related areas in the brain showed a cortical thickening trend, and these areas were not significantly changed after taking their ages into consideration in the model [35], and in a survey included 58 LBP patients, the density of dorsal paracingulate cortex density was found decreased [26]. Some previous studies may reduce or eliminate altered cortical thickness associated with LBP due to an ignorance of controlling important clinical variables (such as a comorbid affective disorder, pain medication, age, or pain phenotypes) [23]. It was showed a thinner brain cortex in the left dorsolateral prefrontal cortex (dlPFC) in CLBP patients, and an increased cortical thickness in the area among 14 patients with a 6-month treatment. Considering multiple factors that may interact with each other and contribute to natural brain alterations, it is necessary to revisit results and theories.

Moreover, grey matter volume alterations occurred in CLBP patients. It was showed that neocortical grey matter volume was 5%–11% less in CBP patients than control ones. And the decreased grey matter volume was associated with pain duration [40]. Similarly, some other studies also observed decreased total grey matter volume, shrinked nucleus accumbens volume [24], and decreased grey matters in the dlPFC and the somatosensory cortex [27, 29, 31, 41], the posterior parietal cortex [42], and the anterior insula (INS) [27, 43]. Some studies showed that regional brain atrophy was linked to LBP, especially on those regions associated with pain processing and modulation, which had a great impact on pain chronicity [44]. Therefore, grey matter density was decreased in brain areas associated with pain processing and modulation and some clinical symptoms (e.g., emotional disorders and memories impairments), including the dlPFC [27, 40], the S1, the thalamus [28, 45, 46], and the middle cingulate cortex (MCC) [28]. However, some studies demonstrated increased volumes in the left ACC and the S1 as well [25]. Thus, whether the consequent grey matter volume is decreased or increased is still in debate. Grey matters in the injured brain were also increased from a decrease after training [47], indicating the change was reversible and curable.

In addition, the functional activity was confined to emotion-related brain regions with acute pain transforming into chronic pain over time [48]. In patients suffering from acute back pain, the activation was concentrated in regions overlapping with pain ones like bilateral INS, the thalamus and the ACC. Meantime in patients with CBP, abnormal activities could be detected in bilateral amygdala and the medial prefrontal cortex (mPFC), which were overlapped with emotional areas [49, 50]. The INS, the ACC, and other sensory-related regions were transiently engaged in spontaneous pain in CBP; however, if the pain remained high, continuous mPFC activity replaced those changes mentioned above whose major function was regulating emotions, responding to conflict, and detecting unfavorable outcomes, especially when they related to oneself, which reflected the intensity of CBP and indicated that emotion related regions were playing their roles [31]. On the other hand, some brain regions like the INS were increasingly activated during acute pain of CBP patients, which tightly reflected the duration of CBP. It was demonstrated a double dissociation between sensory and emotional brain areas when encoding acute pain intensity as opposed to chronic pain [51], which reconfirmed the emotional-related regions were activated corresponding to a long-term back pain. Also, it was showed that one possible way of mental factors and physical ones took effect and feedback in turn. For example, CBP patients suffered from sleep disorders, and sleep disorders resulted in the unpleasant emotions and memories (higher BAI/DOI scores). These further exacerbated the severity of LBP [52]. In comparison with CLBP patients without depression, depressive CLBP ones presented significantly more severe pain and higher levels of pain. These findings provided a new clue connecting physical health and mental health protection. Certain kinds of general psychotherapy may be undertaken into consideration in CLBP patients.

Subjective spontaneous pain of CBP may be associated with specific spatiotemporal neuronal mechanisms, which differs from those observed for acute experimental pain. Hence, it would be likely to determine the course and the duration of back pain and to predict the transition from acute pain to chronic states, especially when dealing with nonspecific patients and patients with symptoms of back pain but actually originated from diseases other than disk degeneration or so. With the aid of imaging approaches, it will be possible to identify various kinds of back pain patients and to keep track on the happening, progression, and outcome of the undergoing diseases rather than appearance symptoms.

### 7.2. Connectivity Alterations

To examine functional connectivities in CBP patients, the resting-state fMRI and task fMRI were applied to measure the spontaneous blood-oxygen-level-dependent (BOLD) activities of brain networks at resting state and the evoked BOLD responses during predefined stimuli individually [22, 53, 54]. There were functional connectives alterations in default mode network through the resting-state fMRI. The left multisensory association area–the left PCC and the left premotor cortex–the left PCC exhibited stronger resting-state functional connectivities in the CLBP patients, whereas two others regions in right primary visual (V1) cortex were decreased functional connectives [21]. With combined resting-state BOLD functional magnetic resonance imaging and 1H-MR spectroscopy, there were significant resting-state functional connectivities to the posterior cingulate cortex and the right anterior INS in salience network and the bilateral hippocampus. An altered balance between the inhibition and the excitation resulted from the functional connectivity of the anterior INS and local neurotransmitter levels [55–59]. And it was worth noticing that the connectivity from mPFC/rACC to related regions varies, showing increase or decrease level of brain function [24, 41, 60]. Also, in the survey done from another perspective, after giving patients manual therapy (MT), using dual regression probabilistic independent component analysis, they found a significant increase in assessed salience network (SLN) connectivity to the thalamus, the primary motor cortex and the lateral prefrontal cortex after therapy, which indicated a reduction of clinical back pain [30, 61]. Previous studies have revealed that the primary somatosensory cortex (S1) played an important role in nociception [62, 63], and the S1 received most somatosensory information from the thalamus [64]. Based on a seed-based analysis strategy, it was showed an increased functional connectivity between the left S1-back and the right superior and middle frontal gyrus (SFG/MFG) [57, 65], indicating a hyperconnectivity of the S1 cortex to both the default mode and executive control networks.

Also, studies indicated that, through therapy like acupuncture [66, 67], manual therapy [68], or so [69, 70], functional connectivity alterations could result in reversion to levels previously reported. These results suggest that connectivity abnormalities were strongly associated with chronic pain pathology in local cortical areas. These changes were reversed to normal standards after gaining treatment (Table 4), which have deepened our understanding. Besides, through comparing the Beck depression score of patients with CLBP at baseline and 6 and 12 months after lumbar discectomy, the situation of concomitant depression symptoms taken place along with the back pain duration appeared to be improved by 6 and 12 months after lumbar discectomy, which supported the intrinsic reasons relying on cortical alterations [34]. Also, further application can be developed in clinic practice, as minor connectivity alterations can be detected if we do regular brain scanning in high-risk populations. We will be able to make early diagnosis, early treatment, and early control of the complicating factor more practical, subsequently reaching a reduction in both government and private health expenditure representing an overall reduction in health expenditure.

## 8. White Matter Alterations

The white matter consists of nerve fibers transmit information among different brain regions to ensure the coordinated operation of the whole brain. With the method of diffusion tensor imaging (DTI), the white matter architecture and integrity were studied in patients with CBP [71, 72] (Table 5). Evidence of alteration of grey and white matter microstructure in the medial prefrontal cortex, the lateral prefrontal cortex, and the nucleus accumbens, along with the connecting fibers had been found with fractional anisotropy (FA) differences, and these changes predicted the transition from acute back pain to chronic pain for patients with a single episode of back pain [74]. In future clinic practice, instead of relying merely on diagnostic criteria to make clinical diagnosis, using scanning technology like DTI, through comparing FA data, physicians will be able to identify early-stage CBP patients from those suffered from acute back pain with those who share similar symptoms, and the reverse is also practical. In a study, it was first confirmed that reduced connectivity to widespread areas and the condition partially recovered after treatment. In order to determine whether a recovery in functional connectivity of the INS was associated with changes in the underlying structures, they used DTI analysis. A comparison was made of the fractional anisotropy (FA) of the white matter of the INS, which was conducted to represent fiber density and myelination [77]. The research showed that, after giving treatment to CLBP patients with decreased FA data in the left INS white matter, the FA data were increased significantly, which confirmed the assumption aroused earlier [69, 75]. And the FA was significantly decreased for CLBP compared to control group in both left and right S1-back regions of interest and the left S1-finger regions of interest [25]. And it is worth noting that a significant reduction in local efficiency in the structural networks of individuals with NSLBP and the left precentral gyrus became significantly more connected [73]. Notably, brain regions for which differences in white matter connection conferred an increased risk for chronic pain using DTI probabilistic tractography-based structural connectivity. They found there was a greater density of white matter connections between nodes concentrated in the corticolimbic area, indicating that increased white matter connections predisposed individuals to chronic pain [76, 78]. And also, as previous work about the grey matter has demonstrated through particular treatments like acupuncture chiropractic spinal manipulation, spinal mobilization, and therapeutic touch, the symptoms along with the alterations in brain can be reversed [67–69]. And such observation offered a new avenue by which we can approach back pain therapy. Same as what have mentioned previously about results of studies on grey matter alterations, these studies contributed to the growing understanding of LBP as well as to improve the diagnosis and treatment protocols.

## 9. Summary

There has been mounting evidence of distinct neuroplasticity and brain remodeling happening in patients suffered from CBP, including grey matter volume and density alterations, functional connectivity alterations, functional activation, and white matter alterations. And these alterations can be detected before the appearance of clinic symptoms, also showing the transition along with the development and outcome of the symptoms. These studies suggest that the quantifying brain changes may serve as important neural indicators for monitoring the development of CBP and evaluating its effectiveness.

Apart from movements disorders, owing to increased brain activities in pain-related brain regions, patients with LBP mostly showed sensorimotor impairments as well as central sensitization. Any level of the ascending pain modulation pathway could amplify the nociceptive signals triggered by external stimuli. Besides pain-related regions in the brain that have been formerly proven played a role in patients suffered from long-duration back pain, many researchers have carried out studies about the part emotion-related nervous system regions, pathways, and networks involved in emotional and cognitive processings. Psychological and cognitive disorders such as depression, anxiety, catastrophizing, and sleep disturbances are common in patients with CBP. We can assume, along with the happening and transition of CBP, regions of interest in the brain connected with emotional processings can be of equal importance. The mechanisms underlying pain negative emotions and pain sensation in CBP patients would elucidate related brain region alterations. Moreover, certain treatment, such as acupuncture, has been testified effectively through not only physical performance but the reverse of former brain alterations scanning by imaging techniques.

In future, there is a need to conduct more neuroimaging studies based on former studies and consequently followed questions to develop a comprehensive and sophisticated understanding of the neural mechanisms and various alterations corresponding to CBP and ultimately to enhance the development of treatment options for CBP management that are more appropriate and effective.

## Figures and Tables

**Table 1:** In- and exclusion criteria.

Inclusion criteria	Exclusion criteria
Experimental studies	Nonexperimental studies
Case-control design	No comparison with healthy controls
Full-text reports	Non-full-text reports
Humans	Animals, infants, children, or adolescents
Presence or history of LBP	Severe LBP pathologies due to cancer, spinal cord injury, or myelopathy
fMRI	Other brain imaging techniques

**Table 2:** Structural alterations of grey matters in back pain patients.

Subjects	Assessed indices	Main findings	Reference
17 patients with nonspecific LBP	Whole brain volume	Ventrolateral prefrontal cortical regions↑	[20]
124 CBP patients	GM cortical thickness	Left PCC and right rostral middle frontal gyrus cortical thickness ↑	[21]
90 CLBP patients	GM volume	Left ACC volume↑, no significant difference in cortical thickness or surface area of the ACC.	[22]
14 CLBP patients	Whole brain volume	No significant clusters of thinning or thickening	[23]
40 SBP patients, 28 CBP patients	GM limbic volume	NAc volume ↓	[24]
103 CLBP patients	GM volume	Primary somatosensory cortex volume↑	[25]
58 LBP patients	GM density	Dorsal paracingulate cortex density↓	[26]
111 CLBP patients	GM density	PFC and the anterior INS density↓	[27]
14 CLBP patients	Total GM volume, total, partial GM volume, GM density	Total grey matter volume ↓, grey matter density in ROI (the PFC and the thalamus) ↓	[28]
18 CLBP patients, 14 of them with 6 months treatment	GM cortical thickness	Left dlPFC cortical thickness before treatment ↓, left dlPFC cortical thickness after treatment ↑	[29]

ACC, anterior cingulate cortex; CBP, chronic back pain; CLBP, chronic low back pain; dlPFC, dorsolateral prefrontal cortex; GM, grey matter; INS, insula; LBP, low back pain; NAc, nucleus accumbens; PCC, posterior cingulate cortex; PFC, prefrontal cortex; ROI, regions of interest; SBP, subacute back pain.

**Table 3:** Studies related to regional activation alterations of grey matters in back pain patients.

Subjects	Assessed indices	Main findings	Reference
20 CBP patients	BOLD activity in intrinsic functional networks	Cortical network activity for the salience network and a local pontine network ↑, network activity in the anterior and posterior default mode network ↓	[30]
24 CBP patients	BOLD activity activation in ROI	Activity in the medial prefrontal cortex ↑	[31]
36 CLBP patients	BOLD activity in cingulo-frontal-parietal cognitive/attention network	Activation in the CFP network ↓, the dlPFC and the dorsal ACC ↓, bilateral SPC in attention-demanding task ↓	[32]
21 patients with CLBP 4 men, 17 women	BOLD activity in brain regions	Activation at the NAc in the CLBP group ↓	[33]
148 CBP patient	Beck depression scores of patients	Depression scores decrease after surgery	[34]
65 CLBP patients	Beck Anxiety Inventory, Beck Depression Inventory and Tampa Scale of Kinesiophobia scores	No association was found between the groups and anxiety and depression	[35]

BOLD: blood oxygenation level dependent; CFP, cingulo-frontal-parietal; dlPFC, dorsolateral prefrontal cortex; NAc, nucleus accumbens; SPC, superior parietal cortices.

**Table 4:** Connectivity alterations of grey matters in back pain patients.

Subjects	Assessed indices	Main findings	Reference
35 CLP patients	FC	A significant resting-state functional connectivity to the posterior cingulate cortex and the right anterior INS in salience network	[55]
124 chronic LBP patients	FC	Multisensory association in two regions in the left PCC↑, two regions in the V1 cortex↓	[21]
14 chronic LBP patients	FC	SLN connectivity to the thalamus and the primary motor cortex ↑ SLN connectivity to the lateral prefrontal cortex ↑.	[61]
40 subacute pian patients, 28 chronic LBP patients	Corticostriatal FC	Functional connectivity of putative LNAc shell to left thalamus ↓, the right and the left NAc ↓, the right caudate and the rACC ↓	[24]
26 LBLP patients	FC	Static functional connectivity between the left S1 back and right superior and middle frontal gyrus (SFG/MFG)↑↑, the left S1chest and the right SFG/MFG↑, the right S1chest and the right SFG/MFG↑, the left S1face and the right MFG↑, the right S1 face and the right inferior parietal lobule ↑	[65]
49 SBP patients	FC in the whole brain	The lateral prefrontal and the parietal cortices ↑, the anterior INS, sensorimotor, and default mode areas↓	[43]
217 LBP patients	FC in the whole brain	DMN FC of the right IPL↓, MD of the thalamus, precuneus and left vPCC↓	[46]
90 CLBP patients	FC in the whole brain	Intrinsic FC between the VTA and its cortical and limbic targets↓, including the bilateral mPFC, rACC, and medial temporal lobe (HIP/PHG) ↓	[41]
14 FBSS patients (persistent back pain)	FC in the salience network, CEN, SeN	FC in the anterior cingulate cortex within the SN↑, medial frontal gyrus in the CEN↑, precentral gyrus within the SeN↑, the medial frontal gyrus in the SeN↓	[50]
Seventeen sex- and age-matched CBP patients, and 32 SBP patients	FC	Extrinsic left hippocampal connectivity ↑, intrinsic bilateral hippocampal connectivity↑	[56]
18 CBP, 19 CRPS, and 14 knee OA patients	FC in the whole brain	The mPFC to the posterior constituents of the DMN↓, to the INS in proportion to the intensity of pain↑	[57]
14 CLBP patients	FC	Connectivity from the INS to widespread TPN and TNN areas↓, the left DLPFC connectivity post-treatment versus pretreatment to connectivity pMCC↑, connectivity to bilateral S1/M1 ↑, connectivity to the right PMC↑, connectivity to the right PPC↑, connectivity to the left temporal↑, connectivity to bilateral fusiform↑, connectivity to bilateral visual ↑	[69]
18 CLBP patients	FC	The PAG and the ventral medial prefrontal cortex (vmPFC)/the rostral anterior cingulate cortex (rACC) ↑	[58]
120 SBP patients	FC in the whole brain	The nucleus accumbens and the prefrontal cortex ↑	[59]
50 CLBP patients	FC in the whole brain	FC between the mPFC/rACC and posterior DMN regions↓ mPFC/rACC FCs↑ the SMN FC between the mPFC/rACC and the precentral (PreCG) and the medial frontal gyri (MFG) ↓ the mPFC/rACC and middle frontal gyrus (MiFG) ↑	[60]

CEN, central executive network; DLPFC, left dorsolateral prefrontal cortex; DMN, default mode network; FBSS, failed back surgery syndrome; FC, functional connectivity; INS, insula; IPL, inferior parietal lobe; LBLP, low-back-related leg pain; LNAc, left nucleus accumbens; MD, medial dorsal nucleus; mPFC, medial prefrontal cortex; NAc, nucleus accumbens; pMCC, prefrontal medial cingulate cortex; PCC, posterior cingulate cortex; PPC, posterior parietal cortex; PAG, periaqueductal grey; rACC, rostral anterior cingulate cortex; ROI, regions of interest; SBPp, subacute back pain persist in having pain; S1 cortex, primary somatosensory cortex; S1 back, the representation of the back in the S1; SeN, sensorimotor network; SLN, silence network; TNN, task-negative network; TPN, task-positive network; V1, primary visual cortex.

**Table 5:** Evaluation of white matter alterations in back pain patients.

Subjects	Assessed indices	Main findings	Reference
14 CLBP patients	Whole brain white matter FA	FA in left INS white matter↑↑, No significant differences in INS right white matter	[69]
17 patients with NSLBP	Global network measures	The structural network values of patients NSLBP ↓↓	[73]
120 SBP patients	Whole brain white matter integrity, Regional white matter	FA values in the left superior longitudinal fasciculus↓, the left retro-lenticular part of the internal capsule↓, part of the corpus callosum including the anterior corona radiata↓	[74]
159 SBP patients	FC in white matter networks	White matter connections between nodes concentrated within the corticolimbic system ↑	[75]
103 chronic LBP patients	S1-adjacent white matter skeleton FA	FA in both left and right S1 ROI, and left S1 ROIs in CLBP↓	[25]
16 CBLP patients	global and regional white matter high intensities	Regional WMH in left hemispheric tracts: anterior thalamic radiation, lower cingulate, inferior longitudinal fasciculus, superior longitudinal fasciculus, and the superior longitudinal fasciculus branch to the temporal lobe	[76]

CLBP, chronic low back pain; FA, fractional anisotropy; NSLBP, nonspecific low back pain; ROI, regions of interest; S1 cortex, primary somatosensory cortex; SBP, subacute back pain; WMH, white matter hyperintensities.

## Data Availability

The data were included within the article.
